# The meaning of dignity for older adults: A meta-synthesis

**DOI:** 10.1177/0969733020928134

**Published:** 2020-07-02

**Authors:** Anne Clancy, Nina Simonsen, Johanne Lind, Anne Liveng, Aud Johannessen

**Affiliations:** 87560 UiT Norges Arktiske Universitet, Norway; Folkhälsan Research Center, Finland; 3855 University of Helsinki, Finland; 87011 University College Copenhagen, Denmark; 6976 Roskilde Universitet, Denmark; Vestfold Hospital Trust, Norway; 11310 University of South-Eastern Norway, Norway

**Keywords:** Dignified care, older adults, invisibility, recognition, meta-synthesis

## Abstract

Dignified care is a central issue in the nursing care of older adults. Nurses are expected to treat older adults with dignity, and older adults wish to be treated in a dignified manner. Researchers have recommended investigating the concept of dignity based on specific contexts and population groups. This meta-synthesis study aims to explore the understandings of dignity from the perspective of older adults in the Nordic countries. Synthesising findings from qualitative studies on older adults’ experiences of dignity has provided important insight into what can be essential for dignified care in a Nordic context. The importance of visibility and recognition for the experience of dignity is an overarching theme in all the studies. The participants’ descriptions mostly implicated an existence dominated by a lack of recognition. The older adults do not feel valued as people or for their contribution to society and strive to tone down their illnesses in an attempt to become more visible and acknowledged as people. Toning down their illnesses and masking their needs can protect their independence. At the same time, becoming less visible can leave them without a voice. The metaphorical phrase *protected and exposed by a cloak of invisibility* is used to express the authors’ overall interpretation of the findings. Lack of recognition and being socially invisible is a genuine threat to older adults’ dignity.

## Introduction

A person’s health and well-being are essential to living as full a life as possible. Older adults are no exception. A good life entails participation in activities and experiences of meaningfulness and inclusion. The World Health Organization^1^ highlights central values, such as participation, justice, equality and independence, as well as the right to health. The word ‘dignity’ denotes ‘respect’ and ‘status’ and is associated with these core values. Dignity is a central theme on the political agenda in all four Nordic countries, namely Norway, Finland, Sweden and Denmark.

In Norway, a regulation called ‘The Dignity Guarantee’^2^ was implemented in 2011. The legislation aims to ensure that older adults are treated with dignity when receiving health and care services.^2^ In Finland, the Act for Elderly Care and Services^3^ came into force in 2013 and, concerning long-term care, states that older people should be cared for in such a way that they can live in dignity and experience their life as safe and meaningful. The dignity policies related to the quality of care for older adults in Denmark focus on the quality of life, autonomy, interdisciplinary and integrated healthcare, food, nutrition, and dignity in death.^4^ The National Board of Health and Welfare in Sweden has adopted a clause to the law on Social Services Act regarding fundamental values in caring for older adults.^5^ These values embrace the right to a dignified life that includes the possibility of experiencing well-being. To experience well-being means to live under secure conditions and to experience an active and meaningful life with others. To live a dignified life entails that social services must be of good quality and that professionals show respect for the older adults’ privacy and integrity. The self-determination, participation and individualisation of older adults must be respected and supported, and caring staff must be responsive and empathetic in their meetings with older adults.^5^

The concept of dignity can be defined as a core value grounded in respect and associated with human rights.^6^ Dignity is also a subjective experience related to autonomy and identity.^6^ Heggestad et al.^7^ emphasise that dignity is not only a theoretical concept but that it has practical meaning and is of importance to older adults, their relatives and healthcare. When experienced in specific situations, dignity seems to be associated with respect, prevailing personal integrity, and with empathic and compassionate caring.^7^

### Dignity in nursing care

Dignity is a central and complex issue in nursing. Nurses are expected to treat older adults with dignity, and older adults wish to not only be treated in a dignified manner but also to die a dignified death.^8–10^ Research indicates that the concept of dignity^11–13^ can be described as *absolute* and *relative.* Absolute dignity relates to fundamental personal freedom and responsibility that is an inherent part of being a person.^6,14^ Relative dignity concerns social and cultural life that can be equally promoted and violated through confirmation from others.^13,15^ Tranvåg et al.^15^ found that to preserve dignity, the experience of confirmation, faith and hope from a loving family is essential. Dignity can be promoted through friendship, and social inclusion and positive relationships with health professionals can confirm the patients’ feelings of self-worth.

Suffering caused by care violates patients’ dignity.^16–18^ Lack of respect for the individual patient leads to distrust between the patient and the caregiver, and feelings of humiliation and inferiority among patients.^19^ Relatives of patients with dementia experience instrumental task-focused care and lack of resources, as opposed to relational and confirming care, as a threat to the patients’ dignity.^7^ The importance of the relational aspect of care to older adults is supported in a recent qualitative literature review.^20^ The review, based on a systematic search protocol and thematic synthesis, found that the key difference between nurses’ and older adults’ perspectives was that older adults emphasised relational aspects, such as trust, relations with other patients, encounters with nursing staff, dependency on others, social network and stigmatisation, whereas nurses highlighted the working culture and environment.^20^ The context for the review was institutionalised nursing care, and 4 of the 14 studies included were from Nordic countries. However, only one of the four studies explored dignity from the older adults’ perspective. The others investigated dignity from the viewpoint of nurses. Though there were similarities, the differences between carers’ and older adults’ perceptions of what is important in care delivery were evident. A recent qualitative study from the Netherlands^12^ showed that the professional caregiver recognises their dignity in the dignity of the person they care for; giving up the one implies no less than giving up the other. The authors concluded that dignity must be understood as relational and that not experiencing dignified care is humiliating for the older adult and also influences the work satisfaction of healthcare staff. Legislation on dignity in the Nordic countries describes the importance of health and care services that preserve the dignity of each older adult.

Compiling, synthesising and interpreting research that has explored older adults’ understandings of dignity in the Nordic countries can provide important insight for future research. The results can guide legislation that supports dignity in healthcare practice. The results can also lead to reflections on if and how healthcare professionals safeguard older adults’ subjective understandings of dignity.

Three decades ago, the Norwegian sociologist and care researcher Kari Wærness^21^ stated in a programme for care research in Norway that researchers have to gain insight into the experiences of individuals. She promoted the importance of bringing forth the voices of those who are in the most vulnerable situations and including their perspectives in health and care research. Gallagher et al.^18^ identified a need for research on dignity regarding different cultural groups.

This meta-synthesis research study, which draws upon a first-person perspective, can provide an important contribution to raising awareness of Nordic older adults’ experiences and understandings of dignity in different settings, with a special focus on healthcare settings, including home care.

## Aim

To explore older adults’ understanding of dignity in different settings in a Nordic context.

## Method

Noblit and Hare’s^22^ meta-ethnographic approach is chosen to synthesise the findings in a sample of qualitative studies on older adults’ understandings of dignity. The goal is to synthesise the results of relevant studies by translating metaphors and key concepts in order to constitute a broad understanding of the phenomenon.^22^ The process of translation, as described by Noblit and Hare^22^ as a process of comparison, was implemented to discover similarities and differences and build a line of argument that can illuminate different aspects of the phenomenon.^23^ Key concepts and metaphors from the findings are compared and contrasted, enabling a new and broad understanding of the phenomenon. Comparison revealed consistencies between the older adults’ accounts across studies. The findings were comparable and did not refute one another. Although the metaphors and key concepts differed as forms of expression, they were analogous in the sense that they promoted a common understanding of the older adults’ experiences of dignity.

Closeness to the primary studies is essential, and quotes are used to support the researchers’ interpretations. France et al.’s^24^ guidelines provide a framework and help ensure that existing recommendations and guidance for conducting and reporting meta-ethnographic studies are followed.

## Data collection

A pilot search was conducted in a multitude of electronic databases in which the keyword ‘dignity’ was combined with *older adults*, *persons*, *experiences*, *perceptions*, *challenges*, *barriers* and *difficulties*. The test search was performed mainly in English, but in Norart and SweMed, search words in Norwegian, Swedish and Danish were used. Finnish language searches were not carried out. The population, interest, context (PICo) tool^25^ helped clarify the research focus and refine the inclusion and exclusion criteria. Table 1 illustrates the process of clarification. The inclusion and exclusion criteria are explicated in Table 2.

**Table 1.** Population, interest, context (PICo).

Population (who)	Interest (what)	Context (where)
Older adults > 65 years	What dignity means to older adults, and their experiences and understandings	Healthcare settings in the Nordic countries

**Table 2.** Inclusion and exclusion criteria.

Inclusion	Exclusion
Primary research articles in English and the Scandinavian languages Research from the Nordic countries Qualitative methodology Research from the Nordic countries that portray older adults’ (aged > 65 years) experiences and understanding of the concept of dignity	Studies that do not have a Nordic perspective Studies that do not include adults aged > 65 years Studies using quantitative methodology Reviews and meta-synthesis that do not represent the first-person perspective

An expert librarian was consulted. The librarian aided the identification of keywords and databases for the search process. The search was performed during January and February 2018. Relevant empirical research articles published in scientific journals from the online databases PubMed (including Medline), EMBASE, PsycINFO, CINAHL, AgeLine, SweMed + and Norart were retrieved. We did not find studies published earlier than 1999 that filled the inclusion criteria. The keywords used for the search process were ‘older’, ‘elderly’ and ‘oldest’, combined with ‘dignity’, ‘experiences’, ‘perception’, ‘challenges’, ‘barriers’, ‘difficulties’ (and ‘qualitative’) in any part of the articles.

**Figure 1.**
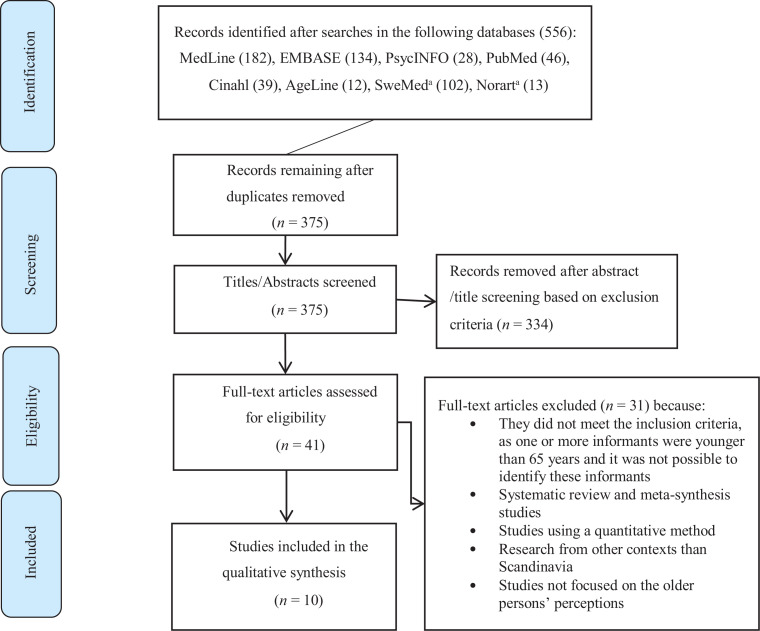
Flow chart of the systematic literature search.^26^ ^a^In SweMed and Norart databases, Norwegian, Swedish and Danish search terms were used.

A flow chart illustrates the inclusion and exclusion process (Figure 1). The systematic search resulted in 556 articles. Duplicates were removed. All titles were screened, and abstracts were then read by the authors. Papers not meeting the inclusion criteria were filtered out during these first stages. Full-text articles were assessed for eligibility, and 31 articles were then excluded (Figure 1). All members of the research team participated in the systematic review process and the appraisal of relevant studies.

## Appraisal of the included studies

The search process and resulting appraisal were carried out by the authors, first individually, then in pairs, and finally, in a group meeting with all five authors present. The process resulted in 10 studies^27–36^ considered suitable for inclusion. The Critical Appraisal Skills Programme (CASP) guide was used. The tool consists of 10 questions that help evaluate the quality of the articles. Table 3 provides an overview of the appraisal process. The CASP tool does not provide a scoring system but appraises the congruity between aims, methods, design, data collection, analysis, findings and discussion for each study.^37^

**Table 3.** CASP, a checklist for appraising qualitative studies.

Number	Study	Qualitative methodology appropriate?	Recruitment strategy appropriate to the aim(s)?	Data collected addressing the research issue?	Relationship between researcher and participants adequately considered?	Ethical issues taken into consideration?	Data analysis sufficiently rigorous?	Clear statement of findings?	How valuable is the research?
1	Andersson et al.^27^	Yes	Yes	Yes	No	Yes, briefly	Yes	Yes	Valuable
2	Axelsson et al.^28^	Yes	Yes	Yes	No	Yes	Yes	Yes	Valuable
3	Bayer et al.^29^	Yes	Yes	Yes	No, not detailed.	Yes	Yes	Yes	Valuable
4	Harrefors et al.^30^	Yes	Yes	Yes	Yes	Yes	Yes	Yes	Valuable
5	Hedelin and Strandmark^31^	Yes	Yes	Yes	Yes, with reference to Hedelin, 2000.	Yes, with reference to Hedelin, 2000.	Yes	Yes	Valuable
6	Heggestad et al.^32^	Yes	Yes	Yes	Yes	Yes	Yes/CT	Yes	Valuable
7	Mangset et al.^33^	Yes	Yes	Yes	Yes	No, not explicitly	Yes	Yes	Valuable
8	Rasmussen and Delmar^34^	Yes	Yes	Yes	Yes	Yes	Yes, however not in depth.	Yes.	Valuable
9	Stikholmen^35^	Yes	Yes	Yes	Yes	Yes	Yes	Yes	Valuable
10	Stabell and Lindström^36^	Yes	Yes	Yes	Yes	Yes	Yes	Yes	Valuable

CASP: Critical Appraisal Skills Programme.

The findings in the included studies were presented in themes or descriptions or both, in or close to the participants’ own words. In the following text, the descriptions and the findings from the included studies are mentioned with numbers. The number of participants in each study varied from 4 participants^34^ to 41 participants.^29^ The characteristics of the 10 included studies are presented in Table 4.

**Table 4.** Characteristics of the included studies.

Author, year, country		Aim	Samples^a^	Context	Analyses	Findings
1.	Andersson et al.^27^	To investigate the experiences of aspects that bring about a good life in the last phase of life when receiving municipal care.	17 (aged 78–100) (10 W/7 M)	Living at home or in special accommodation	Content analysis	An overall theme is: Turning inwards to come to peace with the past, the present and approaching death while being trapped by health complaints. Six categories emerged: Maintaining dignity, enjoying small things, feelings of ‘being at home’, being in the hands of others, trying to adjust, still being important for other people and completing life while facing death.
2.	Axelsson et al.^28^	To describe and to elucidate the meanings of the lived experience of being severely ill and living with hemodialysis when nearing end of life.	8 (3 W/5 M)	Living at home	Phenomenological hermeneutical method	3 themes and 11 subthemes. Being sub ordinate to the deteriorating body. Feeling that fatigue is taking over life Interpreting the deteriorating body Being dependent on others Feeling trapped Having a changing social life Changing outlook of life Living with the sorrow of having to give up plans Having to accept a changed life Hovering between living in the present and worrying about the future Reflecting on the meaning of a life with hemodialysis Striving for upheld dignity Losing control in life with illness Striving to maintain sense of self
3.	Bayer et al.^29^	To explore older adults’ views on dignity and how it is experienced in their everyday lives.	391 persons from 6 countries (283 W/108M) 89 focus groups interviews and 18 individual interviews. 14 focus groups from Sweden (28 W/13 M)	Living alone, with spouses, with other relatives, nursing homes or sheltered accommodation	Inductive thematic analysis	Three overarching themes were identified and described; Respect and recognition Dignity in care, participation and involvement. The researchers found close connection between dignity and suffering.
4.	Harrefors et al.^30^	To describe older people’s perceptions of how they wanted to be cared for in the future.	12 couples (aged > 70)	Living at home	Content analysis	Central is the importance of being able to maintain the self and being cared for with dignity to the end. Three categories were created: to be at home as long as possible when partners can take care of each other, to have professional care at a nursing home when care is needed, and fear of being abandoned when extensive care is needed.
5.	Hedelin and Strandmark^31^	To gain a deeper understanding of the meaning of mental health by investigating how elderly women perceive their own mental health.	12 W (aged 71–92)	Living at home or service flats in the municipal	Phenomenological approach	Confirmation is the core constituent of mental health. Confirmation – that is the perception that one is noticed, respected and regarded as a valuable person – is the cornerstone for zest for life, trust and confidence in the future, development and involvement in one´s relationship to oneself and to others.
6.	Heggestad et al.^32^	To present and discuss what persons with dementia themselves experience as a threat to their dignity.	6 (aged 84–94) 5 W/1M	Two nursing home units	Content analysis	Residents feel that their freedom is restricted and describe feelings of homesickness. They experience that they are not being seen and heard as autonomous persons. Both may be a treat to their personal dignity. Summary of findings: to maintain a person’s dignity, it is important that the person is confirmed as an individual person.
7.	Mangset et al.^33^	To identify factors contributing to elderly stroke patients’ satisfaction with rehabilitation following stroke.	12 (7 W/5M) One male < 65	Living at home or stroke rehabilitation unit	Systematic text condensation	The overall category is that patients wants to be treated with respect and dignity. The five subcategories exemplifies what this means: To be treated with humanity, being acknowledged as an individual, having one’s autonomy respected, having confidence and trust in the professionals and having dialogue and exchange of information.
8.	Rasmussen and Delmar^34^	To describe characteristics of the importance of dignity perceived by four surgical patients and understand the lifeworld and the informants’ experiences.	4 (2 aged > 60)	Vascular surgery unit	Phenomenological approach	The researcher stresses the findings of a dilemma about dignity. On the one hand it is natural to overstep boundaries of privacy and there is an understanding with fellow patients who help each other to maintain dignity. However, the informants’ stress information about care and treatment from nurses is of major importance to dignity. So fellow patients cannot stand alone if dignity is to be maintained.
9.	Stikholmen^35^	To shed light on how elderly patients’ need of maintaining own experience of dignity is expressed during admission to hospital	9 W (aged 70–99)	Three hip fracture units	Systematic text condensation	Respondents’ need to maintain own sense of dignity was expressed via values they felt important to live by. Values were independence, roles of responsibility and caring, and orderliness. Being allowed to express the values, having them reaffirmed and being helped to live by them supported the patients in maintaining dignity. Healthcare professionals must respond to these requests.
10.	Stabell and Lindström^36^	To explore in what way residents in a nursing home keep up their feelings of integrity and dignity.	5 (aged 80–96) Gender unknown	Three wards in one nursing home	Hermeneutic approach	Elderly people in increasing need of other people’s help are vulnerable to loss of dignity and integrity. The residents adopt in different way to their new situation and change their values in order to experience control. They compensate old values with new ones or enhance existing values. The staffs’ interaction pattern plays a crucial role in the process.

^a^Gender; Woman = W: Men = M; 2 391 persons from 6 countries (283W/108M), included in the study. Eighty-nine focus group interviews and 18 individual interviews were performed, and 14 of the focus groups were performed with Swedish participants. 3 One of the participants in study 7 and two participants in study 8 were < 65.

### Contextual information

According to France et al.,^24^ it is important to provide contextual information that illustrates how the included studies are related. The older adults in the 10 included studies share thoughts on what promotes and what inhibits dignity but also speak about their strategies for maintaining dignity. The participants lived at home or in assisted caring facilities (Table 4). Some had chronic illnesses that required hospitalisation, and their specific experiences were related to hospital stays.^33–35^ It was described as being easier to define the absence rather than the presence of dignity.^3^ Being marginalised and devalued were given as examples of the absence of dignity in all the included studies, whereas being seen, respected and confirmed, experiencing belonging and having some freedom of choice were all associated with an experience of dignity.

## Analysis

### The process of analysis: Synthesis and meta-synthesis

Following the eMERGe guidelines,^24^ the findings from each study were read carefully. The authors paid close attention to both similar and divergent findings in order to gain insight into older adults’ experiences of dignity in healthcare settings. Main concepts and metaphors were compared across studies to determine if they were similar or in opposition to each other. This strategy facilitated establishing a line of argument for presenting the findings. The comparison of findings across studies led to the creation of new themes that promoted a novel and comprehensive understanding of the phenomenon of dignity. Finally, a metaphorical phrase was created that captures the essence of the findings.

## Process of translating the studies

The goal of meta-ethnography is to develop a more comprehensive understanding of personal experiences.^24^ The studies were sufficiently similar to enable a reciprocal translation analysis of the findings across studies. Noblit and Hare’s^22^ meta-ethnographic approach was used when comparing the findings. The purpose was to make sense of similarities (analogous) or differences (refutational) and to develop a line of argument that could provide a deep understanding of older adults’ experiences of dignity in the Nordic countries. In this approach, metaphors, themes, concepts and contexts were compared across studies. The authors were careful to pay attention to all findings so that similarities and inconsistencies were considered. Possible alternative interpretations were discussed in team meetings during the translation process. The authors strived for transparency throughout the study. Steps were taken to remain close to the first-person perspective by including detailed descriptions from the primary studies.

Examining the older adults’ statements and the use of metaphors caused the emergence of new themes that had relevance across studies. Creative interpretations can enhance a comprehensive understanding of the phenomenon.^38^ The final process involved a further creative synthesis at a meta-level.

Metaphors, symbols and similes from the findings are compared and contrasted, enabling a new and broad understanding of the phenomenon. Metaphors convey meaning^39^ and have the power to affect our conception of reality.^40^ Symbols are understood as representing an idea, whereas similes call attention to something similar.

## Findings

Based on the interpretation of the findings in the 10 studies (Table 4), the following four themes emerged: (1) *An ailing body and mind – a threat to dignity?* (2) *The fear of becoming a nobody – the need to be acknowledged;* (3) *The importance of participation and capabilities;* (4) *Being a victim, feeling trapped.*

## An ailing body and mind – a threat to dignity?

Maintaining dignity entailed keeping up a façade of normality, and although health complaints affected the older persons’ lives, they did not complain and tried to conceal their symptoms from others.^27,28^ Although they wanted to trust health professionals, they were worried about losing their autonomy and independence^28,29^ if they became too dependent on them. Experiences of dignity were closely related to suffering.^27–29,36^ An 89-year-old woman spoke about toning down her illness in order to maintain a sense of whom she was:^27^

I don’t complain to my sons, telling them that I’m in pain, no, that doesn’t help anyone…But I am in pain.

The participants struggled to have control over their bodies and illnesses.^28^ In order to retain a sense of self, they spoke about their former strengths and tried to be active, despite their limitations. Besides, they were very susceptible to feelings of uselessness and were especially vulnerable if healthcare professionals showed a lack of interest in their situation.^28^ It was easy to feel excluded and ignored,^32^ and they stressed the importance of being acknowledged.^28,29,35^ Dignified treatment was important: Being pain-free, having clean clothes, a clean bed, as well as privacy and human contact, were all central to dignity.^29^

When patients perceived incidents of indignity, they interpreted it as the staff having degraded them as human beings.^29,33^ One woman, aged 72 years, explained:^33^

It was when I wanted to go to the toilet. And I couldn’t manage at all. And I asked. ‘No, you’ve just been to the toilet’. ‘But, oh dear, I’ll do it in my pants’, I said. So, she said: ‘Well I couldn’t care less’.

Respecting privacy was important.^34^ Patients felt that their dignity was violated when curtains between the beds were not pulled across, or they felt exposed in multi-bed wards, and when other patients could hear conversations between professionals and patients. A woman aged > 69 years expressed her fear of being abandoned:^30^

I do not know what the worst is, but if you are in good mental health and realise you are totally dependent on care, it seems so horrible, then it must be better to be lost of mental capacity.

When the body is deteriorating, the illness or lack of functions must not define the older person.^36^ To be treated with dignity and respect and maintaining a sense of self are crucial to being satisfied with care.^30,32,33^ To be treated with dignity entails being treated with humanity, being acknowledged as an individual and having one’s autonomy respected. The feeling of being acknowledged and feeling worthy of the staff’s attention influenced patients’ satisfaction:^33^ ‘It means a lot that you feel you exist and aren’t being ignored’ (Woman, 87 years). On the contrary, when patients/residents experienced that the staff degraded them as human beings, it threatened their sense of self-worth:^33^ ‘They are very insensitive, aren’t they? We’re just sick people, nothing else’ (Woman, 77 years).

The staff holds power to either confirm or ignore the older person. The women who participated in the study^31^ could not experience trust and confidence or zest for life if they did not experience confirmation of their worth in their relationships with others. When older adults were asked about their future needs, the desire to maintain their sense of self and to be treated as a unique person seemed to become more important the more vulnerable and the more in need of care they became.^30^

## The fear of becoming a nobody – the need to be acknowledged

The fear of becoming a nobody and the need to be acknowledged were a central theme in all the studies. The older adults were concerned about not being seen as individuals, being disregarded, and they worried about becoming a nobody and lacking meaningful relations with others.^27,30,31^ A 91-year-old woman spoke about her worst fear: ‘That I should become a living wreck’.^28^ Encounters with others can be perceived as confirming when older adults, in this case, older women, feel that they are seen as people.^31^ However, they could feel objectified, controlled and depersonalised by health professionals during hospital stays.^28^ As one 74-year-old woman said:^34^

…Some say you have to go to bed before the nightshift arrives. I didn’t like that. Then, I would toss and turn. That is not respect. They have to respect my daily rhythm because I only sleep [for] 5 hours.

Older adults with dementia are especially vulnerable and dependent on the healthcare staff to listen to their wishes and needs. As one of the older adults explained: ‘when you are not listened to, there is a feeling of not being confirmed as an autonomous person’, which confers an experience of a threat to one’s dignity.^33^ The older adults spoke about the importance of being able to maintain a sense of self.^30^ By talking about themselves, their view of life and their history, they formed a conception of themselves as valuable people.^31^ Having a zest for life and having a positive attitude towards oneself and good relationships with others were important and confirmed human existence and dignity.^31^

Poor communication practices were mentioned as a threat to dignity: using pet names, such as *Dear* or *Love* or being ignored.^29,33^ The participants^28,34^ stressed the importance of being listened to and acknowledged as central to their experience of dignity. A Swedish woman spoke about a wheelchair-user being completely ignored; all communication was ‘carried out over her head’.^29^

Being dependent on others meant that they lost control and could be suspicious that information was being withheld from them. The participants were afraid of being a nuisance:^28^ ‘And you don’t want to go on nagging because you don’t want to be regarded as a, a…nag’ (Woman > 65 years). Another older person spoke about the importance of being recognised:^34^ ‘It is difficult when you are not allowed to be who you are; I take pride in doing what I can’ (Woman, aged 70 years).

Being seen, respected and regarded as people worthy of respect from others was important.^29,31,32,34^ Their feelings of self-worth were important, providing strength and a sense of value.^27,31^ Older persons in a residential home adapt to their new situation in different ways but feel that they have little control over their lives.^36^ However, when patients are given choices and invited by the professionals to be involved in care and treatment, they do feel they are in control and that their dignity is maintained.^34^

Lack of control seems to threaten the older person’s sense of being respected.^33^ The older adults wanted to be accepted by the nurses, and some spoke of positive experiences.^28^ Others worried about situations where they were among people who did not know their needs, their life story or the type of care they desired.^30^ They spoke of the importance of being cared for by someone who recognised their physical, psychological and spiritual needs.^30^

## The importance of participation and recognition of capabilities

Despite being aware of their impending death, participation and having a role in other people’s lives was vital for the older adult’s sense of dignity.^27^ Being at home meant that they could experience a sense of security in familiar surroundings and have control of their daily lives.^27^ They were conscious not to show how sick they felt and not to be a burden to others but instead to be a part of a context consisting of family and friends.^27^ However, if they trusted the professionals,^27^ then feelings of being at home could also be experienced in institutions. A caring environment that promotes trust can provide feelings of control, security and dignity.^27,29^ They valued being fully informed about decisions related to their care,^29^ as information made the patients feel important and capable of participation.^29,34^ It is the quality of the way care, whether informal or formal, is provided, rather than where the care is provided that is central.^27^ Care must promote control, security and dignity.^27^ Both women and men emphasised the importance of living with their partner as long as possible.^30^ Being together meant that they could support and advocate for each other.

The older adults expressed feelings of exclusion.^27,29^ Depreciation with increasing age denied older adults ‘a useful role’. Loss of independence was a major concern. As one Swedish participant said:^29^

As long as you can manage on your own and not be a bother to someone else…then I think you live a dignified life. (Gender not specified >65 years)

To be respected for the roles and identity one previously had in the family provides proudness and dignity.^35^ One way the older women confirmed their human existence and dignity was by talking about their life and history, bringing up children, doing handcrafts, and, in that way, confirming themselves as people with distinctive characteristics, living in specific contexts and having a mission in life.^31^ The women had all achieved something that they were able to value.^31^ They also described the will and ability to promote their health and well-being and the courage they needed to safeguard their integrity and autonomy. One woman spoke about standing up for herself:^31^

I thought I was very bold when I visited my doctor and told him I didn’t want his help anymore. I mean, if you go to a doctor and you’re much worse when you leave, then he’s not suitable. It’s funny because I got so bloody angry; I was almost recovered when I left the place. (Woman, >70 years)

The importance of being seen and heard, and being taken seriously as a person, was mentioned.^30^ The participants stressed the importance of co-determination and participation in their care and treatment, as long as the disease had not taken their strength and power.^34^

Respect, recognition and participation from others and for one’s self, as well as being included and recognised, were important.^29^ Other participants had experiences of not having their autonomy respected, as the healthcare staff did not acknowledge and value their knowledge, skills and experiences.^33^ As one woman, aged 77 years, said:

‘Cause they boss you around, ‘cause you’re sick, aren’t you? And then you’ve got no say. I’ve noticed that now.

However, not all older adults wanted to be involved in decision-making regarding their medical condition and felt it could be frustrating.^33^ To be in control was a balancing act between withholding information from the professionals to retain their right to make decisions and showing confidence and leaving decisions to the professionals.^28^

## Being a victim, feeling trapped

When thinking about future needs, there was a fear of being isolated, without their partner and friends, and of having to spend hours alone waiting for someone to come.^30^ All participants in the study^30^ expressed a fear of being trapped, with no human relations and facing horrible loneliness.

The respondents in one study^32^ lived in a nursing home. They expressed that their freedom was restricted and that they were not treated as autonomous people.^32^ Lack of freedom and not being confirmed and recognised as individual autonomous people were a threat to their self-dignity. Their dependence on help to get out and about made them feel like captives.^32^ One woman (aged 86 years) said:

You know it is like a prison without bars (…) I feel like a prisoner. I have no freedom.^32^

This account was in contrast to descriptions of being at home.^32^ One 82-year-old woman said:

[A home is]…a place where you may walk around and do what you like. If I want to walk in the garden, I can do so, and if I just want to sit down and read a book, I can do so. And I don’t have to be afraid of what others think about what I am doing […].

Other residents in care facilities felt that they had to adjust and fit in, so as not to disturb the routines.^27,35^ Chronically ill older adults felt trapped in their ailing bodies. Increasing fatigue also left them feeling excluded.^28^ However, in contrast, there were also descriptions of the acceptance of their ailing bodies. As one 84-year-old woman said:

I don’t think ‘how terrible’. I’ve never used such a word…If I say that I have followed the tide, followed life, it has been like that year after year.^31^

The patients experienced that the longer time they spent in the hospital, the less freedom they had to live their lives.^28^ They experienced an increasing dependency on both family and caregivers and that they were burdens that left them feeling vulnerable and frustrated.

## Meta-synthesis of findings — ‘protected and exposed by a cloak of invisibility’

The metaphorical phrase *protected and exposed by a cloak of invisibility* is used to express the authors’ overall interpretation of the findings. The importance of visibility and recognition for the experience of dignity is an overarching theme in all the studies. Expressions such as being ‘a nobody’ and ‘feeling trapped’ inferred associations with invisibility, non-existence and lack of freedom. The metaphors, symbols and similes used in the studies show that the older adults do not always positively describe themselves. Metaphors are associated with the experiential context and with physical and cultural experiences.^40^ The participants in the study use the following metaphors to describe their situation: ‘swallow bitter pills’, ‘a living wreck’, ‘a nag’. To *swallow a bitter pill* suggests something unpleasant and difficult, a situation that is prescribed by others and beyond their control. *A living wreck* can imply suffering and signify a useless object that exists but no longer functions. A *nag* can be considered both a metaphor and a simile, as it denotes both an *old horse* and an annoying person. *Follow the tide* was also used as an expression of how they had become used to their situation over the years and just went along with it.

The participant’s descriptions implicated an existence dominated by a lack of recognition. The older adults do not feel valued as people or for their contribution to society. Lack of recognition and being socially invisible is a genuine threat to their dignity. They strive to tone down their illnesses in an attempt to become more visible and acknowledged as persons. Their ailing bodies were seen as a threat to their dignity. Making their illnesses and vulnerabilities invisible in order to protect their integrity and independence is interpreted as a strategy to promote their own and others’ awareness of their self-worth.

Older adults are indignant when they are not involved in decision-making. Exhibiting anger and disappointment can be ways of expressing that their dignity is threatened. Sarvimäki and Stenbock-Hult^41^ describe how a quest for freedom and responsibility can often be followed by experiences of pain and suffering. Resistance is, however, a sign of hope and courage to live.^41^

Recognition is fundamental for dignity.^42^ Society’s prevailing attitude towards older adults as burdens is evident in the older adults’ stories. Honneth^42^ describes how a lack of recognition plays a huge role when people feel wrongly treated and disrespected. Not being recognised limits the older adults’ sense of freedom and creates feelings of being trapped and useless. In order to boost their self-confidence, the older adults used different strategies to maintain their dignity and promote their self-respect. The strategies entailed either standing up for themselves with the risk of being regarded as *annoying nags* or just going with the flow and toning down their needs and illnesses. By expressing their needs, they ran the risk of losing control over their lives and receiving negative responses.

Anchored in anthropology and philosophy, Honneth^43^ defines recognition as a basic human need that is crucial for self-confidence, self-respect and self-esteem. Social acknowledgement takes place in an interpersonal space – as human beings, we maintain a positive relationship with ourselves through confirming relationships with others. Repeated experiences of being treated with disrespect can result in the older adult’s feeling the need to avoid conflict. Going under the radar to avoid being noticed seemed to be an alternative for the participants in the study. When they experience their situation as hopeless, becoming invisible is seen as a valid strategy to avoid conflict and protect themselves from unpleasant situations. According to Honneth,^42^ social invisibility is detrimental to a person’s inherent dignity^43^ and is a form of disrespect.^42,43^ Honneth^43^ argues that there is a difference between the cognitive awareness of another person and recognition. Making a person visible entails recognising this person as a unique individual. Conversely, becoming invisible can leave the older adult vulnerable and exposed. To be erased by society is one aspect, but if this results in the older adults erasing themselves, then they can become invisible and their needs as people ignored. Metaphorically speaking, there is then no one to hold the pen and no paper on which to make their mark. The findings in this study reveal that we must continue to focus on dignified practice. According to Gallagher et al.,^18^ nursing is about recognition and response to claims of worth. In the authors’ view, it is also vital that nurses recognise and respond to claims of worthlessness.

## Methodological reflections, strengths and limitations

Synthesising findings from qualitative studies on dignity in Nordic countries provides important insight into older adults’ views, experiences and understanding of dignity in a Nordic context. Preserving the original meanings from the included studies and creating a meta-synthesis provide a comprehensive understanding of the phenomenon of dignity that extends beyond the findings in each study. The meta-synthesis expressed in the metaphorical phrase *protected and exposed by a cloak of invisibility* creates a framework for interpretation. A meta-synthesis requires a secondary analysis of primary studies that facilitates a higher level of interpretation.^38^ The process is always influenced by the meta-synthesis researchers’ personal reflections in both primary and secondary studies. The authors are aware of the danger of over-interpretation of findings. The authors have sought transparency throughout the study and provided contextual information and quotes from the primary studies. We followed the guidelines for conducting and reporting meta-ethnographic studies^24^ so that the research process was as transparent as possible.

Although an experienced librarian carried out the searches, some relevant studies might not have been found. The included studies were, however, primarily from Sweden and Norway, with one from Denmark and none from Finland. Cultural norms can influence how dignity is experienced, and the findings from one study may not be representative in all contexts. Moreover, the majority of the participants in the studies were females. Thus, the synthesis is limited in capturing gender-specific issues. However, the included studies were from different settings – homes, nursing homes, hospitals and wards for people with dementia.

## Recommendations and conclusion

Synthesising the included qualitative studies provided the research team with more insight into older adults’ experiences and understandings of dignity in different settings in a Nordic context. It can be pertinent to ask if older adults in the Nordic countries are recognised and respected. This meta-synthesis has shown that being regarded as a person to be reckoned with is essential for experiencing dignity. Health and social care professionals are educated to meet the needs of older adults. Legislation on dignity does not ensure respectful care if dignity is not implemented as a value in health care settings. The older adults do experience dignified care, but their stories mostly portray a lack of recognition and confirmation of their worth and contribution to society. Being considered old can put the older adult at risk of being interpreted in a certain way and ignored.

Further gender-specific studies on dignity should be carried out, as well as studies that consider social class and educational levels. Further comparative research from different countries can illuminate whether diverse environments and policies for the care of older adults influence their experiences of dignity. Intervention studies in healthcare settings that aim to promote care that advocates dignified care would also be worthwhile.

More focus is needed on social recognition in education and healthcare. Healthcare professionals need to recognise that being *a nag* or *a nuisance* can be a sign of courage and necessary resistance to unhealthy power structures in healthcare settings. Older adults need to be cared for by professionals who recognise not only their needs but also their concerns and continued contribution to society. Being treated with respect can promote a sense of security and enable the older person to be honest about their vulnerability and limitations. This can, in turn, give them a sense of freedom and hope for the future.
